# Harvester ant seed removal in an invaded sagebrush ecosystem: Implications for restoration

**DOI:** 10.1002/ece3.6963

**Published:** 2020-10-29

**Authors:** Kelsey E. Paolini, Matthew Modlin, Alexis A. Suazo, David S. Pilliod, Robert S. Arkle, Kerri T. Vierling, Joseph D. Holbrook

**Affiliations:** ^1^ Haub School of Environment and Natural Resources University of Wyoming Laramie WY USA; ^2^ Department of Fish and Wildlife Sciences University of Idaho Moscow ID USA; ^3^ Department of Forest, Rangeland, and Fire Sciences University of Idaho Moscow ID USA; ^4^ U.S. Geological Survey, Forest and Rangeland Ecosystem Science Center Boise ID USA

**Keywords:** cheatgrass, desert granivore, *Pogonomyrmex*, sagebrush restoration, seed removal

## Abstract

A better understanding of seed movement in plant community dynamics is needed, especially in light of disturbance‐driven changes and investments into restoring degraded plant communities. A primary agent of change within the sagebrush‐steppe is wildfire and invasion by non‐native forbs and grasses, primarily cheatgrass (*Bromus tectorum*). Our objectives were to quantify seed removal and evaluate ecological factors influencing seed removal within degraded sagebrush‐steppe by granivorous Owyhee harvester ants (*Pogonomyrmex salinus* Olsen). In 2014, we sampled 76 harvester ant nests across 11 plots spanning a gradient of cheatgrass invasion (40%–91% cover) in southwestern Idaho, United States. We presented seeds from four plant species commonly used in postfire restoration at 1.5 and 3.0 m from each nest to quantify seed removal. We evaluated seed selection for presented species, monthly removal, and whether biotic and abiotic factors (e.g., distance to nearest nest, temperature) influenced seed removal. Our top model indicated seed removal was positively correlated with nest height, an indicator of colony size. Distance to seeds and cheatgrass canopy cover reduced seed removal, likely due to increased search and handling time. Harvester ants were selective, removing Indian ricegrass (*Achnatherum hymenoides*) more than any other species presented. We suspect this was due to ease of seed handling and low weight variability. Nest density influenced monthly seed removal, as we estimated monthly removal of 1,890 seeds for 0.25 ha plots with 1 nest and 29,850 seeds for plots with 15 nests. Applying monthly seed removal to historical restoration treatments across the western United States showed harvester ants can greatly reduce seed availability at degraded sagebrush sites; for instance, fourwing saltbush (*Atriplex canescens*) seeds could be removed in <2 months. Collectively, these results shed light on seed removal by harvester ants and emphasize their potential influence on postfire restoration within invaded sagebrush communities.

## Graphical abstract

Plant diversity and abundance is profoundly influenced by granivores and is particularly important for degraded landscapes, such as the sagebrush‐steppe. Here, we examine the factors influencing seed removal by Owyhee harvester ants. We found abiotic and biotic processes driving seed removal, and apply removal rates to historical sagebrush restoration treatments to determine the potential impact in restored landscapes.

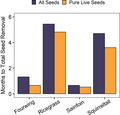

## 1 INTRODUCTION

Seed availability drives plant biodiversity (Chambers & MacMahon, 1994) and is often influenced by strong bottom‐up processes, particularly in desert ecosystems with limited and patchy resources (e.g., water, soil nutrients, organic matter; Cammeraat & Risch, 2008; Crist & Wiens, 1993). Within arid landscapes, scarcely distributed resources and plant dispersal mechanisms generate fragmented plant communities (Brown et al., 1979). Plant distribution is further modified by top‐down pressures—specifically, seed removal. Desert granivores structure plant communities through both seed removal and active nutrient cycling (Bachen et al., 2018; Gosselin et al., 2016; Maron et al., 2012). For example, banner‐tailed kangaroo rats (*Dipodomys spectabilis*) are mound‐building granivores capable of altering soil properties, wildlife presence (e.g., lizards), and plant composition (Edelman, 2012; Schooley et al., 2000). Indeed, seed removal influences plant distributions and abundance, and may indirectly favor invading exotic plants in the presence of strong seed selection on native species by granivores (Lucero & Callaway, 2018; Pearson et al., 2011; White & Robertson, 2009).

Given the potential impact of desert granivores to ecosystem‐level processes, a more complete understanding of the role of seed removal in structuring plant communities could help ecologists understand historical and ongoing environmental changes, as well as improve restoration outcomes. Arid communities are experiencing transformative disturbances including desertification, tree encroachment, and extensive wildfire that are increasingly atypical of desert fire regimes (Balch et al., 2013; Bestelmeyer et al., 2015). Global desertification, for example, reduces landscape heterogeneity and resource heterogeneity, leading to expansive shifts in plant community structure and composition (Bestelmeyer et al., 2015; Schlesinger et al., 1990). Throughout western North America, big sagebrush (*Artemisia tridentata*) communities also face multiple pressures contributing to large‐scale changes, including degradation from urban development, livestock grazing, and perhaps, most notably, are alterations in wildfire regimes (Balch et al., 2013; Coates et al., 2016; Kepner et al., 2000; Prevéy et al., 2010). Non‐native cheatgrass (*Bromus tectorum*) has steadily invaded sagebrush ecosystems for decades and, as a fire‐prone species, creates a cheatgrass–wildfire cycle. In this cycle, degraded sagebrush systems are susceptible to invading cheatgrass, which increases wildfire frequency, further facilitating cheatgrass establishment; this process often leads to human interventions in the form of extensive and expensive restoration efforts (Barker et al., 2019; Brooks et al., 2004; Chambers et al., 2014). Sagebrush restoration techniques include protecting native plant communities, preventing non‐native invasion, and restoring degraded areas (Chambers et al., 2014). Given the expansive changes resulting from the cheatgrass–wildfire cycle within the sagebrush ecosystem, coupled with other pressures such as livestock grazing and observed changes in altered precipitation patterns (Miller et al., 2011), evaluating the magnitude and importance of seed removal in this plant community could help improve restoration outcomes that often experience limited success (e.g., Knutson et al., 2014).

A number of studies have assessed seed removal within desert environments, where granivorous small mammals and ants place strong pressures on seed availability (Anderson & MacMahon, 2001; Bachen et al., 2018; Reichman, 1979). In the absence of granivores, sagebrush communities increased in species richness, plant density, and soil nutrients, signifying the importance of granivores in shaping community structure (Maron et al., 2012). Desert granivores, although often seed generalists, exhibit preferences that can shift in response to landscape disturbance and seed availability (Lucero et al., 2015; MacMahon et al., 2000). For instance, desert rodents and ants altered seed consumption when foraging in burned or unburned desert scrub environments (Suazo et al., 2013). Fire disturbance depletes seed banks resulting in costly restoration treatments (James et al., 2011; Knutson et al., 2014), which are additionally subjected to selective seed removal by granivores. Although previous work has advanced our understanding on the role of granivores in desert systems (e.g., Lucero & Callaway, 2018; Suazo et al., 2013), much remains unknown concerning seed removal by small mammals and ants in the context of biological invasion and restoration in sagebrush‐steppe ecosystems. Improving our understanding with respect to granivore seed selectivity and removal can help direct future management with the goal of successfully restoring invaded sagebrush ecosystems.

The main aim of our study was to characterize seed removal and determine the ecological factors influencing seed removal for a desert granivore, the Owyhee harvester ant (*Pogonomyrmex salinus* Olsen), in a highly degraded, sagebrush‐steppe ecosystem that has undergone frequent burning by wildfires, cheatgrass invasion, and restoration. Harvester ants modulate foraging behaviors in response to seed availability and traits (e.g., energy content; Crist & MacMahon, 1992; Lucero et al., 2015), while successful restoration also depends on seed availability. Our first objective was to assess harvester ant selectivity for four plant species commonly used in sagebrush restoration. We predicted harvester ants would select seeds that balance high nutrient content with an energetic trade‐off for handling time (Brown et al., 1979). Harvester ants also exhibit temporal activity patterns, becoming dormant in winter months (Kwapich & Tschinkel, 2013) leading to cessation of seed harvest. Our second objective, therefore, was to determine monthly removal for each seed species, while harvester ants were active, to evaluate the potential effects of ants on sagebrush restoration efforts. Microclimatic conditions within sagebrush communities also drive harvester ant foraging. For example, temperatures alter daily foraging activity which can be geographically dependent (Stuble et al., 2013, 2014); however, harvester ants generally display unimodal activity in cooler months and bimodal patterns in warmer months (Crist & MacMahon, 1992; Stuble et al., 2013, 2014). Vegetation structure, resource distance, and biotic factors, such as larger colonies and neighboring nests, are additional factors that can influence harvester ant foraging (Anjos et al., 2019; Gordon & Kulig, 1996; MacMahon et al., 2000). Our final objective was to evaluate the relative effects these factors had on seed removal by harvester ants. We expected higher seed removal during the middle range of temperatures within an active period. We predicted an increased prevalence of cheatgrass and distance to seeds would lead to declines in removal, largely because of increased search and handling times as seeds radiate away from the nest in dense vegetation. We also predicted higher seed removal by harvester ants under the context of additional exploitative pressures from larger nests located in close proximity (MacMahon et al., 2000). This work not only advances the understanding of seed removal within biologically invaded landscapes, but also provides information that can be used to inform plant restoration efforts.

## 2 MATERIALS AND METHODS

### 2.1 Study area

The Morley Nelson Snake River Birds of Prey National Conservation Area (NCA) is a 1,962 km^2^ region in southwestern Idaho (Latitude: 43.283, Longitude: 116.200). The NCA has a semi‐arid climate (110–350 mm annual precipitation; 5.4–19°C average annual temperatures) and was historically dominated by sagebrush‐steppe plant communities and, to a lesser extent, salt‐desert scrub. The land is managed by the U.S. Bureau of Land Management (BLM) under a multiple land use framework, including part of the land area that is comanaged by the Idaho Army National Guard for combat training. Much like other parts of the northern Great Basin, fire frequency has increased on the NCA because of persistent invasion by non‐native grasses and forbs (Pilliod et al., 2017). Conversion of sagebrush and salt‐desert shrublands to annual grasslands, primarily dominated by cheatgrass and postfire rehabilitation seedings conducted by BLM (e.g., Pilliod et al., 2017), has created a gradient in vegetation communities and successional stages. Shrub communities include big sagebrush (*Artemisia tridentata*) and winterfat (*Krascheninnikovia lanata*). Primary grasses include native perennials (e.g., fourwing saltbush, *Atriplex canescens*), non‐native perennials used as part of postfire restoration treatments (Russian wildrye, *Psathyrostachys junceus*), and non‐native annual grasses (cheatgrass). The study area had sparse native forbs and abundant non‐native forbs, particularly bur buttercup (*Ceratocephala testiculata*), prickly Russian thistle (*Salsola tragus*), clasping pepperweed (*Lepidium perfoliatum*), and tall tumblemustard (*Sisymbrium altissimum*). In addition to harvester ants, there are at least eight small mammals that are common and forage on seeds within our study area, such as the Great Basin pocket mouse (*Perognathus parvus*), Ord's kangaroo rat (*Dipodomys ordii*), and western harvest mouse (*Reithrodontomys megalotis*; Baun et al., 2010).

### 2.2 Owyhee harvester ant and vegetation sampling

Our questions aimed to evaluate how a gradient of cheatgrass invasion influenced harvester ant foraging within sagebrush‐steppe communities. We focused on cheatgrass‐invaded communities because of its extensive distribution across the western United States and abundance within our study area. To assess our questions, we sampled 11 0.25 ha plots from 6 June 2014 to 9 July 2014. At each plot, we used aboveground photography to sample 9 grid‐point intercept quadrats arranged on a 25 m grid to characterize vegetation characteristics (Pilliod & Arkle, 2013). Photographs were taken 2 m above the ground, resulting in a 1.5 × 2 m area of ground per photograph. We used SamplePoint 1.43 software (Booth et al., 2006) to measure bare ground and cheatgrass canopy cover at 100 computer‐selected grid points (i.e., pixels) on each image. Average cheatgrass canopy cover ranged from 40% to 91% across our sampled plots, which was our primary gradient of interest.

Within each plot, we censused and mapped active harvester ant nests. To accomplish this, we divided the plots into 25 × 25 m quadrants and systematically surveyed each plot by walking transects spaced 5 m apart. We walked transects in both a north to south and east to west direction to expose nests concealed by vegetation. Any nests outside of the plot, but within 14 m of the plot boundary, were recorded to account for the maximum foraging distance observed by harvester ants in a similar environment (Crist & MacMahon, 1992). This allowed us to evaluate questions associated with nearest nest for those on the plot boundary and determine whether potential intraspecific competition influenced seed removal.

**Figure 1:**
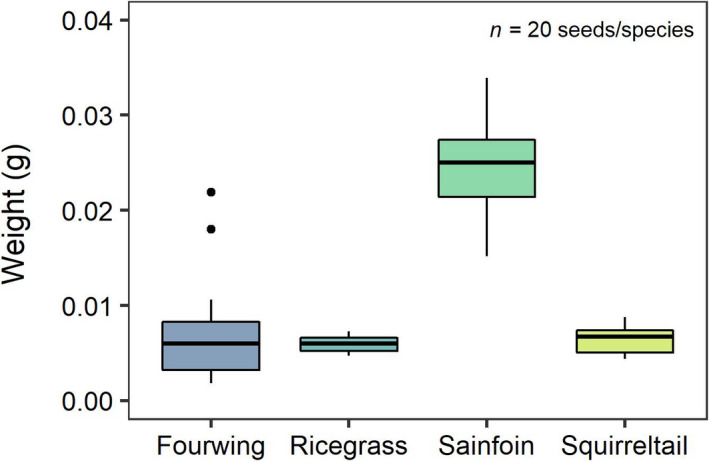
Relative seed weights for plant species presented along Owyhee harvester ant (*Pogonomyrmex salinus*) foraging trails. Thick horizontal lines represent median values (i.e., 50th percentile), the lower and upper limits of the shaded boxes represent the 25th and 75th percentiles, respectively, and dots display outliers

At each identified nest, we presented seeds obtained from the BLM Boise District Office from four plant species: fourwing saltbush (native), bottlebrush squirreltail (*Elymus elymoides*; native), Indian ricegrass (*Achnatherum hymenoides*; native), and sainfoin (*Onobrychis viciifloia*; non‐native). We presented these species because they are commonly used in restoration treatments by the BLM and vary in seed weight (Figure 1). We did not present cheatgrass seeds to (1) focus our questions on how harvester ants influence sagebrush restoration efforts, and (2) limit the additional spread of cheatgrass in our study area.

For each nest, we identified the discrete path the majority of ants were foraging along, commonly referred to as the trunk or trail, and recorded the trail azimuth. Although harvester ants forage up to 15 m from the nest, foraging intensity increases closer to nests (Crist & MacMahon, 1992; Ostoja et al., 2013; Willard, 1963). Therefore, we placed two daily pill organizers on either side of the foraging trunk at 1.5 m and 3.0 m from the edge of the nest disk (i.e., the area directly adjacent to the nest that has been cleared of vegetation by ants). Each pill organizer contained a systematic arrangement of 20 seeds per species within a single compartment, resulting in 40 seeds presented per species per distance (a total of 80 seeds per species per nest). Through a series of pilot experiments, we found 20 seeds per compartment, regardless of species, were sufficient to prevent complete seed removal at either distance. Harvester ants had access to seed compartments through holes drilled in the side of each pill compartment and flush with the ground. Nails through the two exterior compartments secured boxes to the ground surface. We taped the lids of all pill boxes closed prior to placement to avoid seed removal from diurnal nontarget granivores, such as Piute ground squirrels (*Urocitellus mollis*).

We recorded the duration of harvester ant foraging during the 2‐ to 4‐hr morning foraging period (approximately 0900–1200) at each nest once during our study. At the end of the morning foraging period, we collected pill organizers and counted the number of seeds remaining, which allowed us to calculate seed removal for each monitored nest. To calculate an average temperature during foraging activity, we recorded hourly temperatures at the ground surface using Onset ® HOBO ® pendant temperature loggers with a solar radiation shield.

After monitoring morning foraging activity and seed removal, we measured additional attributes associated with the nest. We inserted pin flags into the nest and measured from the ground, not including debris, to the top of the nest to estimate nest height (cm), which can indicate colony age and size (Scott, 1951); however, nest height may be indicative of soil conditions (e.g., Folgarait, 1998). We also measured distance to the nearest nest using the proximity tool in ArcMap 10.1, which may be used as an index of potential exploitative competition (Howell & Robertson, 2015).

### 2.3 Data analyses

#### 2.3.1 Seed selection

We evaluated differences in seed selection (Lele et al., 2013) among the four plant species presented using proportion tests to determine whether harvester ants actively selected certain species relative to all presented seeds (Altman et al., 2013; Wilson, 1927). The test for equality of proportions indicated harvester ants exhibited differences in seed selection, and we subsequently conducted pairwise comparisons for each species pair within distances. We adjusted *p*‐values for pairwise comparisons with a Bonferroni correction (Holm, 1979) to account for increasing type I error with multiple comparisons.

#### 2.3.2 Monthly seed removal and Restoration impacts

To characterize monthly seed removal at the plot level (i.e., 0.25 ha plot), we first calculated the total hourly seeds removed for each nest within the plot and averaged across all nests to compute a mean hourly seed removal rate. Applying the observed temperature range associated with morning foraging activity at each plot (range = 20–40°C) allowed us to estimate the potential number of hours per day harvester ants foraged over our experimental period (*n* = 34 days). We then calculated daily seed removal at the plot level based on daily temperatures within the active foraging range. We accounted for nest density by multiplying the number of foraging hours per day for each plot by the average seeds removed per hour and density of nests within the plot. To estimate monthly seed removal by harvester ants for varying nest densities, we multiplied daily seed removal by 30 days.

To determine the potential impacts of harvester ant seed removal on restoration sites across BLM managed lands, we used our monthly removal estimates to identify the length of time in which harvester ants could deplete the average density of seeds used in restoration treatments. Using the Land Treatment Digital Library historical database (Pilliod & Welty, 2013), we calculated the average amount of seeds per 0.25 ha applied in restoration treatments for each of our presented seed species (i.e., fourwing saltbush, Indian ricegrass, sainfoin, and bottlebrush squirreltail) across the western United States. To ensure seed availability was scaled appropriately, we transformed seeds per acre to the plot level (i.e., 0.25 ha; Table 1). Seed applications included aerial seeding techniques, and the amount of seed was calculated based on the total amount of seeds and the amount expected to germinate (i.e., pure live seeds). We used our estimated monthly seed removal for sites with 15 harvester ant nests per 0.25 ha to estimate the amount of time for harvester ants to completely remove species from restoration sites, assuming consistent foraging activity and exclusive harvest. Our values were likely conservative (e.g., max of 15 nests per 0.25 ha) in that other studies have documented up to approximately 40 harvester ant nests per 0.25 ha (MacMahon et al., 2000).

**Table 1:** Restoration information on the seed species (Pilliod & Welty, 2013) presented along foraging trails for Owyhee harvester ants (*Pogonomyrmex salinus*)

Common Name	Genus	Total Acres Seeding BLM 1980–2010	Average Pounds/Acre	Average Seeds/Pound	Average Seeds/0.25 ha
Fourwing Saltbush	*Atriplex*	968,294	1.4	45,617.17	39,427.15
Indian Ricegrass	*Achnatherum*	1,269,431	1.38	191,079.09	162,432.76
Sainfoin	*Onobrychis*	823,501	1.16	27,644.89	19,767.28
Bottlebrush Squirreltail	*Elymus*	1,623,371	1.16	195,296.27	140,487.00

Note

Total acres refers to the sum of all areas in which known seeding application occurred for a particular plant species on BLM managed lands in the Great Basin from 1980 to 2010.

The average seeds per 0.25 ha represent all seeds deployed.

#### 2.3.3 Environmental influences on seed removal

Finally, we created a series of models to test our hypotheses and predictions concerning the number of seeds removed by Owyhee harvester ants. We used mixed‐effects Poisson's models that included our variables of interest (i.e., temperature, cheatgrass canopy cover, bare ground, distance from nest, distance to nearest nest, and nest height) as fixed effects to evaluate our predictions. We assessed multicollinearity among covariates and found that all variables were uncorrelated (variance inflation factors < 3; Zuur et al., 2010). To compare the relative effect sizes, we standardized our covariates by subtracting the mean and dividing by the standard deviation (Gelman, 2008). Each model within our candidate set contained the same baseline structure, treating nest as the sampling unit. To account for differences in the amount of time spent foraging per nest, each model contained an offset, which was the log of minutes during an observation period for a particular nest. We treated nest as a random effect in our models to account for variation among nests and distances (1.5 and 3.0 m) using the *lme4* package in R (R Core Team, 2020). We used the Gauss–Hermite quadrature technique to approximate likelihood, which is generally more accurate than the Laplace approximation, although more computationally taxing (Bolker et al., 2009).

To compare models, we used Akaike's information criterion adjusted for small sample size (AIC_c_; Burnham & Anderson, 2002); we considered models supported if ΔAIC_c_ was < 2. We report model weights for each of these models. To further evaluate model fit for our entire candidate set, we calculated Spearman's correlation coefficient (ρ) between our modeled predictions and observed values. We conducted all analyses in R.

## 3 RESULTS

**Figure 2:**
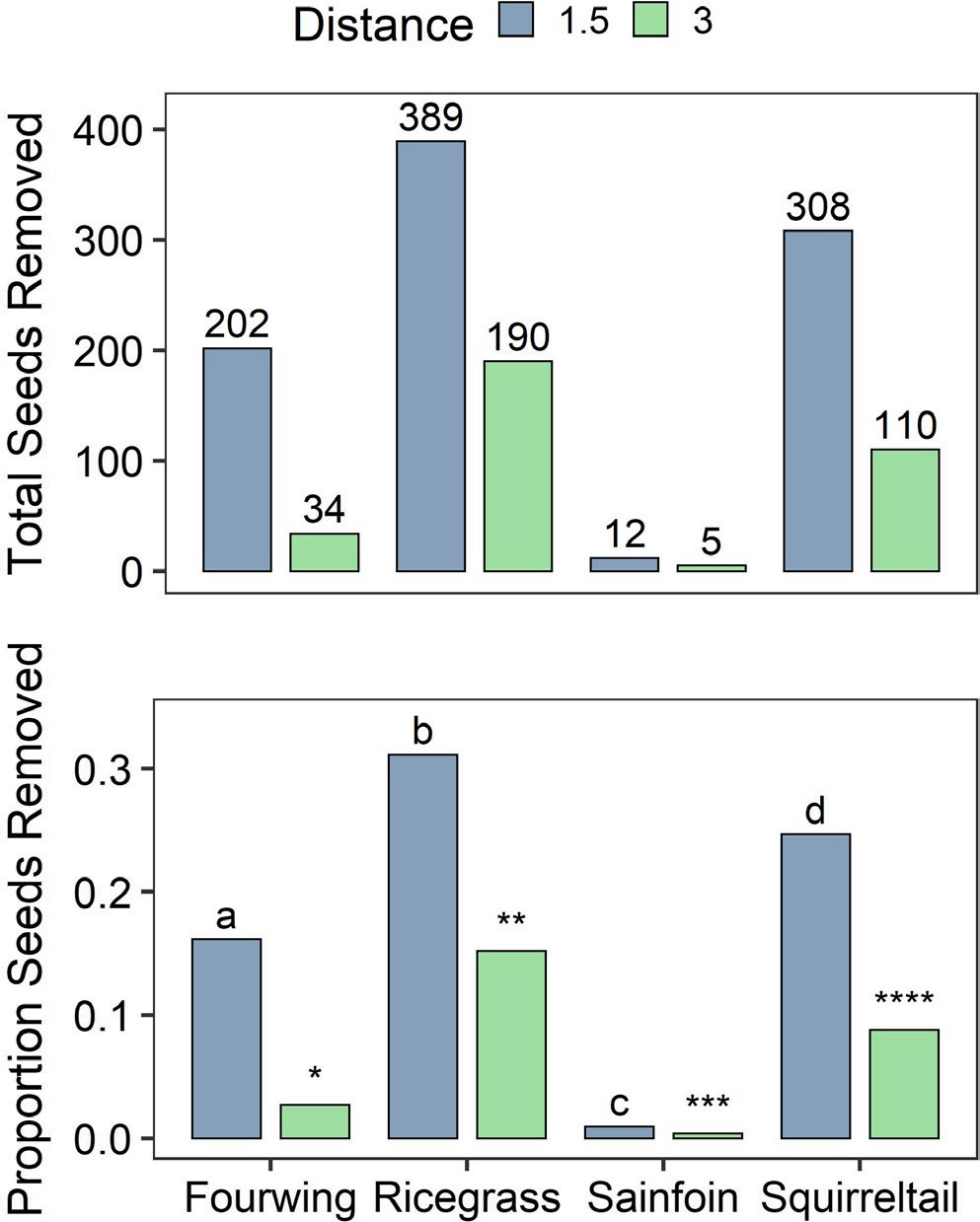
Seed selection by Owyhee harvester ants (*Pogonomyrmex salinus*) when presented with seed species along foraging trails at 1.5 and 3 m from the nest. Letters and asterisks (*) denote significant differences (Bonferroni‐corrected *p*‐values <.001) for pairwise comparisons *within* distances

We identified and sampled a total of 76 harvester ant nests across the 11 plots. We discovered differences in seed selection among our four plant species at both 1.5 m (*Χ* ^2^ = 428, *df* = 3, *p* < .001) and 3 m (*Χ* ^2^ = 261, *df* = 3, *p* < .001). Harvester ants exhibited the strongest selection for Indian ricegrass (46%), followed by bottlebrush squirreltail (34%), fourwing saltbush (19%), and the weakest selection for sainfoin (1%), the only non‐native restoration species we tested (Figure 2). All pairwise comparisons among species at both distances were statistically significant (all Bonferroni‐corrected *p*‐values < 0.001).

The average seeds removed per hour per nest (i.e., combined across seed species and distances) was 5.42 seeds (95% CI = 3.48–12.58 seeds). Estimated daily foraging time differed across plots, ranging from 10.82 to 14.32 hr. Density of harvester ant nests varied from 1 to 15 nests (median = 9.0 nests) per 0.25 ha plot. Combining average hourly seed removal together with hours of potential foraging time and nest density resulted in an estimated daily seed removal of 63 seeds (range = 40–145 seeds) for a 0.25 ha plot with one nest, and 995 seeds (range = 638–2,307 seeds) for a 0.25 ha plot with 15 nests. These values resulted in a monthly removals of 1,890 seeds (range = 1,200–4,350 seeds) for a plot with a single nest and 29,850 seeds (range = 19,140–69,210 seeds) for a plot with 15 nests.

**Figure 3:**
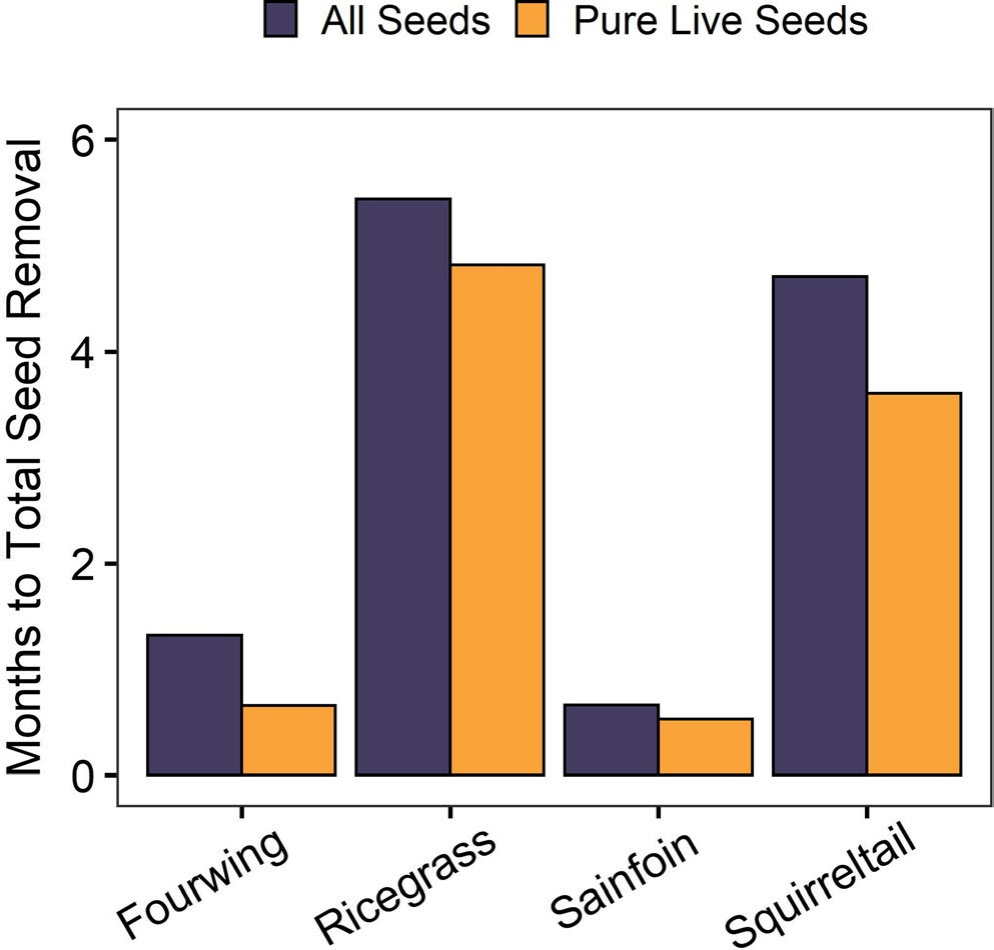
Number of months until all restoration seeds could be removed by Owyhee harvester ants (*Pogonomyrmex salinus*), assuming our maximum observed colony density (i.e., 15 nests per 0.25 ha), observed monthly seed removal (29,850 seeds per month), and exclusive harvest for each species by harvester ants. “All seeds” indicates the time needed to remove 100% of the total number of seeds deployed. “Pure live seed” represents the time needed to remove 100% of *viable* seeds sown (i.e., those expected to germinate)

Comparing the average seeding rate for each plant species with our projected monthly seed removal estimates from harvester ants, and assuming exclusive harvest for each species, our results suggest harvester ants have the potential to remove entire seed applications within 0.5 to 8 months (Figure 3; Table 1). Harvester ants are generally active in our study area from April to October, meaning selective pressures on specific species could diminish seed treatments during this time frame. Interestingly, the least selected species from our removal experiments (i.e., fourwing saltbush, sainfoin) show the fastest time to total removal (Figure 3); however, the average number of seeds planted within a 0.25 ha restored area are an order of magnitude less than those of Indian ricegrass or bottlebrush squirreltail (Table 1).

Our modeling results provided substantial support for a single, top model (i.e., all other models had ΔAIC_c_ > 2) to explain environmental factors influencing harvester ant seed removal. This model contained temperature, cheatgrass cover, nest height, and distance to seeds. Coupling our top model with the second‐ and third (i.e., global model)‐ranked model captured 93% of the Akaike's weights through the addition of distance to nearest nest and bare ground (Table 2); however, the effect sizes were not statistically different from zero. Our null model was among the lowest ranked, suggesting that our explanatory variables and ecological hypotheses were informative. Spearman's correlation coefficients between our predicted values and observed data generated values indicating our models fit the data well (Table 2).

**Table 2:** Hypotheses and model selection results for mixed‐effect Poisson's models assessing removal of Owyhee harvester ants (*Pogonomyrmex salinus*)

Model	*K*	AIC_c_	ΔAIC_c_	*w*_i_	ρ
Temp + Temp^2^ + Dist + Cheatgrass +Nest Height	7	780.2	0.00	0.59	0.79
Temp + Temp^2^ + Dist + Cheatgrass +Nest Height + Nearest Nest	8	782.3	2.10	0.21	0.79
Temp + Temp^2^ + Dist + Cheatgrass +Bare Ground + Nest Height + Nearest Nest	9	783.2	2.97	0.13	0.79
Temp + Temp^2^ + Dist + Cheatgrass +Bare Ground + Nest Height + Nearest Nest + Cheatgrass*Dist	10	784.6	4.37	0.07	0.79
Temp + Temp^2^ + Dist + Cheatgrass	6	788.3	8.06	0.01	0.79
Temp + Temp^2^ + Dist	5	793.6	13.42	0.00	0.79
Dist	3	799.1	18.93	0.00	0.78
Cheatgrass	3	1,062.2	282.04	0.00	0.77
Nest Height	4	1,063.2	238.03	0.00	0.77
Temp + Temp^2^	3	1,064.7	284.49	0.00	0.77
Null	2	1,068.8	288.59	0.00	0.77
Bare Ground	3	1,070.1	289.90	0.00	0.77
Nearest Nest	3	1,070.8	290.63	0.00	0.77

Note

Each model structure contained an offset for minutes (i.e., log minutes) of observation and a random intercept of nest. The number of estimated parameters and Akaike weights for each model are represented by *K* and *w*_i_, respectively. Spearman's correlation coefficients (*ρ*) indicate approximate model fit.

Variable abbreviations are as follows: Dist, distance of seeds removed; Temp, temperature; Temp^2^; quadratic term for temperature.

**Figure 4:**
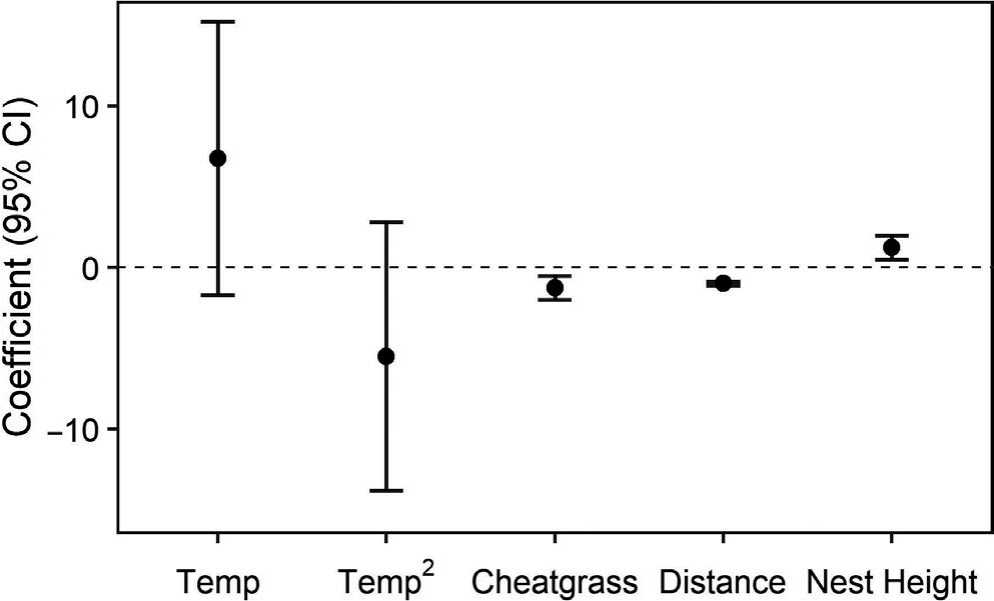
Standardized regression coefficients from our top model characterizing how environmental covariates influenced seed removal by Owyhee harvester ants (*Pogonomyrmex salinus)*. Variable abbreviations include: Temp—temperature; Temp^2^—quadratic term for temperature; distance refers to seed distance from nests (i.e., 1.5 m, 3.0 m)

Our top model indicated Owyhee harvester ants removed fewer seeds when cheatgrass was more prominent in the landscape (Figure 4). Seed removal also declined as foraging occurred farther from the nest. We found a significant positive relationship between nest height (range = 0 – 18 cm) and seed removal. Taller nests, and presumably older and larger colonies, correlated with higher seed removal compared to younger and smaller colonies. The effect of temperature was variable and statistically insignificant (temperature range = 20.0 – 39.7 ℃; Figure 4).

## 4 DISCUSSION

Despite the recognized importance of desert granivores in structuring plant communities, few studies have evaluated seed removal within biologically invaded landscapes such as the sagebrush ecosystem. Here, we evaluated multiple questions concerning seed removal from a desert granivore, the Owyhee harvester ant, in an invaded landscape of the sagebrush‐steppe. We discovered harvester ants selectively removed species with lower relative weights, which is consistent with other studies (Azcárate & Peco, 2006; Suazo et al., 2013). Seed removal deteriorated with cheatgrass abundance and distance from nests, whereas taller nests and presumably larger colonies were associated with increased seed removal. Our estimated daily and monthly seed removal by harvester ants demonstrated a remarkable capacity for ants to impact seed availability within sagebrush communities, particularly when nests are densely aggregated. Moreover, our findings suggest harvester ants could quickly diminish seeds following seeding treatments on BLM lands, which is particularly concerning given harvester ants are one of at least nine granivores in our study area. However, it is important to note this assumes consistent seed removal and exclusive harvest during months in which harvester ants are foraging.

We found that multiple environmental characteristics influenced harvester ant seed removal in a biologically invaded ecosystem. Consistent with our predictions, seed removal declined with greater cheatgrass cover and when seeds were located farther away from the nest (i.e., 3.0 m compared to 1.5 m). Invasive cheatgrass grows more densely and continuously than native bunchgrasses (e.g., Indian ricegrass), likely adding search and handling time for ants to acquire resources (Hickey et al., 2016; Ostoja et al., 2013; Radnan et al., 2018). Although cheatgrass may appear to protect native restoration species from removal, harvester ants avoid removing non‐native cheatgrass seeds which could prove costly for sagebrush restoration (Robertson & Robertson, 2020; Schmasow & Robertson, 2016). Similarly, we found distance‐dependent seed removal, with lower removal at farther distances suggesting restoration may be more successfully applied when distanced from harvester ant nests. However, seed placement was concentrated along foraging trails and removal effects may be reduced as seeds are dispersed in more natural conditions. Distance‐associated removal by harvester ants is likely context‐dependent, where the existing plant community and seed availabilities impact foraging (Ostoja et al., 2013). Although we could not directly quantify harvester ants consuming seeds from the existing plant community, introducing our experimental seeds simulates a rapid influx of resources that occurs when applying restoration treatments and provides insight into the impacts of granivory across gradients of cheatgrass‐invaded sagebrush.

Our results suggest taller nests were associated with more seed removal in sagebrush‐steppe. Within invaded sagebrush communities, harvester ants constructed nests in open areas and nest density, but not occupancy, was higher in postfire restoration landscapes (Holbrook et al., 2016; Robertson & Robertson, 2020). Using remote sensing techniques to measure the effects of invasive species spread in sagebrush‐steppe landscapes is becoming increasingly more pressing (Reinhardt et al., 2020). Previous studies used aerial mapping to assess harvester ant nest density and found wide‐ranging densities and distributions relative to vegetation structure (Crist & Wiens, 1993). Applying recent technological advancements in light detection and ranging (LiDAR) that allows for fine‐scale digital elevation models could be used to calculate nest height and densities (e.g., Anderson et al., 2018), and help identify regions that may require control of ant populations while simultaneously monitoring the long‐term effectiveness of restoration treatments (e.g., Glenn et al., 2016).

Large‐scale environmental changes in restored sagebrush communities include fluctuating ambient temperature (Del Toro et al., 2015). Temperature initiates harvester ant foraging activity (MacMahon et al., 2000), but we found no significant effects of temperature on seed removal within the foraging period. We suspect this is due to sampling within the foraging period versus sampling the gradient of temperatures throughout the day, which could help capture the full range of temperatures that facilitate seed removal by harvester ants. Generally, harvester ants exhibit single activity peaks during cooler months and bimodal peaks in hotter months to prevent reaching lethal body temperatures (Crist & MacMahon, 1991; MacMahon et al., 2000). Additional research with longer temporal sampling that fully captures foraging activity would provide a more precise estimate of total daily and monthly seed removal by harvester ants across their active season (e.g., April–October).

Granivore selection for seeds used in restoration has important implications for the successional dynamics in degraded sagebrush ecosystems. When presented with species commonly used in sagebrush restoration efforts, harvester ants actively removed more seeds of Indian ricegrass and bottlebrush squirreltail, the species’ with the least variability in relative seed weight (Figure 1). Both plant species prevent wind erosion, yet each has distinct successional properties; Indian ricegrass matures slowly, taking up to five years to fully establish whereas bottlebrush squirreltail matures within two years (Tilley et al. 2006; Ogle et al., 2013). Furthermore, bottlebrush squirreltail is a fire‐resistant competitor of non‐native cheatgrass (Tilley et al. 2006). Harvester ants removed lesser amounts of fourwing saltbush and sainfoin seeds. Fourwing saltbush exhibits similar qualities to Indian ricegrass in crude protein content (13%–14%), time to establishment, and wind erosion control (Howard, 2003; Tirmenstein, 1999), indicating this species could be a potential alternative in restoration treatments to help prevent removal by harvester ants. Harvester ants removed minimal sainfoin seeds. Although a large‐seeded species with high crude protein content that is beneficial for browsers (Lauriault et al., 2003), sainfoin takes two to three years to mature (Pyke et al. 2018). Deploying sainfoin together with other species more heavily selected by harvester ants could therefore promote species with faster establishment, such as non‐native cheatgrass. It is important to note we only presented a few plant species and recognize harvester ant selectivity could change as a result of relative seed availability (Schmasow & Robertson, 2016; Wilby & Shachak, 2000). Our results, however, suggest certain species may need to be added into restoration treatments at higher volumes to compensate for seed removal by harvester ants, which would likely increase application costs. Here, we found harvester ants preferentially selected for both early‐ and late‐successional restoration species, which could indirectly aid the cheatgrass–wildfire cycle by reducing restoration success. Nevertheless, additional work examining seed selection and removal of the full suite of granivores is needed to precisely inform plant restoration efforts.

Large‐scale disturbances alter granivore behavior, which ultimately can influence the spatial heterogeneity of plant communities within the sagebrush‐steppe. In fire disturbed arid environments, for example, granivorous rodents and ants varied seasonal seed removal (Suazo et al., 2013) and fire disturbance facilitated higher nest densities of harvester ants (Holbrook et al., 2016; Ostoja et al., 2009). We found that densely aggregated nests (i.e., 15 nests per 0.25 ha) were correlated with an extensive capacity for seed removal on a daily and monthly basis, removing roughly 30,000 seeds a month (range = 19,140–69,210 seeds) and potentially contributing to removal of common restoration species. Other studies have also found high seed intake rates by harvester ants, ranging from 2,700 to 48,000 seeds removed monthly by three nests (Crist & MacMahon, 1992). Although we focused on selective pressures from harvester ants, mammalian desert granivores often prefer native species placing additional constraints on native plant establishment and thus potentially allowing for cheatgrass spread (Lucero et al., 2015; Lucero & Callaway, 2018; Ostoja et al., 2013; Suazo et al., 2013). Because both rodents and ants avoid consuming cheatgrass seeds, our observed effects of removal on restorative seeds could intensify when multiple granivores are sympatric (Anderson & MacMahon, 2001). Additional work could examine top‐down effects from granivory on sagebrush restoration, while recognizing selective pressures may shift based on resource competition by granivores and fluctuating seed availabilities.

Comparing seed removal from harvester ants to average seed availability from restoration treatments showed harvester ants could greatly impact the success of restoring degraded sagebrush communities. While the estimated amount of time for seed removal assumes our highest observed nest densities and that harvester ants concentrate on a single species (Figure 3, Table 1), our objective was to understand the greatest potential impact of harvester ants on restoration efforts. The most removed species (i.e., Indian ricegrass, bottlebrush squirreltail) by ants had higher volumes of seeds in restoration treatments leading to longer times until complete removal, which could extend past the time frame harvester ants are active. This is particularly important for restoration planning where harvester ant behaviors can result in costly removal of restoration plants (Robertson & Robertson, 2020). While we specifically assessed seed removal by harvester ants, restoration treatments also face additional pressures where larger seeded species may experience higher risk of removal from the larger mammalian granivores (Lucero & Callaway, 2018; Maron et al., 2012). Granivores in restored landscapes certainly alter plant species composition and could further deplete seeds available for collection and future implementation in restoration (e.g., Jones, 2019). However, it should be noted that harvester ants can also serve as ecosystem engineers, where foraging behaviors (e.g., altering soil nutrients or vegetation removal) may indirectly benefit native plants, as found in restored Mediterranean grasslands (De Almeida et al., 2020) and sagebrush ecosystems (Gosselin et al., 2016). Ecosystem engineering from harvester ants is an important consideration for sagebrush management and restoration.

Successfully restoring degraded ecosystems depends upon matching seeded species to climate and soil conditions present at restoration sites, as well as anticipating future landscape change. Distinct seed selectivity for, and removal of, restoration plants by harvester ants demonstrated the substantial impact ants can have on restoration efforts in disturbed landscapes. Indeed, effectively restoring sagebrush communities may require planting additional seeds to compensate for granivory pressures. Harvester ants are one of a suite of granivores contributing to seed removal, further indicating the importance of granivores on the long‐term structuring of plant communities through top‐down processes. Collectively, these results emphasize one facet of the complexities of restoring degraded sagebrush communities, which may benefit from a comprehensive approach that includes both the long‐term direct and indirect effects of granivores.

## CONFLICT OF INTERESTS

The authors declare no conflict of interest.

## AUTHOR CONTRIBUTION

**Kelsey E Paolini:** Conceptualization (equal); Data curation (equal); Formal analysis (equal); Investigation (equal); Methodology (equal); Project administration (equal); Visualization (equal); Writing‐original draft (equal); Writing‐review & editing (equal). **Matthew Modlin:** Conceptualization (equal); Data curation (equal); Formal analysis (equal); Funding acquisition (equal); Investigation (equal); Methodology (equal); Project administration (equal); Supervision (equal); Visualization (equal); Writing‐original draft (equal); Writing‐review & editing (equal). **Alexis A Suazo:** Conceptualization (equal); Data curation (equal); Funding acquisition (equal); Investigation (equal); Methodology (equal); Project administration (equal); Supervision (equal); Visualization (equal); Writing‐review & editing (equal). **David S Pilliod:** Conceptualization (equal); Data curation (equal); Funding acquisition (equal); Investigation (equal); Methodology (equal); Project administration (equal); Supervision (equal); Visualization (equal); Writing‐review & editing (equal). **Robert S. Arkle:** Conceptualization (equal); Data curation (equal); Funding acquisition (equal); Investigation (equal); Methodology (equal); Project administration (equal); Supervision (equal); Visualization (equal); Writing‐review & editing (equal). **Kerri T Vierling:** Conceptualization (equal); Data curation (equal); Funding acquisition (equal); Investigation (equal); Methodology (equal); Project administration (equal); Supervision (equal); Visualization (equal); Writing‐review & editing (equal). **Joseph Holbrook:** Conceptualization (equal); Data curation (equal); Formal analysis (equal); Funding acquisition (equal); Investigation (equal); Methodology (equal); Project administration (equal); Supervision (equal); Visualization (equal); Writing‐original draft (equal); Writing‐review & editing (equal).

## Data Availability

Data are available in the Dryad Digital Repository, https://doi.org/10.5061/dryad.sf7m0cg4b.
